# Cleavage of natural rubber by rubber oxygenases in Gram-negative bacteria

**DOI:** 10.1007/s00253-023-12940-3

**Published:** 2024-02-02

**Authors:** Tulika Prakash, Sandhya R. Yadav, Marius Bürger, Dieter Jendrossek

**Affiliations:** 1 School of Biosciences and Bioengineering, Indian Institute of Technology (IIT), Mandi, HP 175005 India; 2 Institute of Microbiology, University Stuttgart, Stuttgart, Germany

**Keywords:** Natural rubber, Poly(*cis*-1,4-isoprene), Rubber oxygenase, RoxA, RoxB, RoxC, Haem, Dioxygenase, NR-degrading consortia, *Aurantibaculum cummioxidans* 35Y

## Abstract

**Abstract:**

Bacterial degradation of natural rubber (NR) in an oxic environment is initiated by oxidative cleavage of double bonds in the NR-carbon backbone and is catalyzed by extracellular haem-containing rubber oxygenases. NR-cleavage products of sufficiently low molecular mass are taken up by the cells and metabolized for energy and biomass formation. Gram-negative and Gram-positive NR-degrading bacteria (usually) employ different types of rubber oxygenases such as RoxA and/or RoxB (most Gram-negative NR-degraders) or latex clearing protein Lcp (most Gram-positive NR-degraders). In order to find novel orthologues of Rox proteins, we have revisited databases and provide an update of Rox-like proteins. We describe the putative evolution of rubber oxygenases and confirm the presence of a third subgroup of Rox-related proteins (RoxCs), the biological function of which remains, however, unclear. We summarize the knowledge on the taxonomic position of *Steroidobacter cummioxidans* 35Y and related species. Comparison of genomic and biochemical features of strain 35Y with other species of the genus *Steroidobacter* suggests that strain 35Y represents a species of a novel genus for which the designation *Aurantibaculum* gen. nov. is proposed. A short summary on the capabilities of NR-degrading consortia, that could be superior in biotechnological applications compared to pure cultures, is also provided.

**Key points:**

*• Three types of rubber oxygenases exist predominantly in Gram-negative microbes*

*• S. cummioxidans 35Y contains RoxA and RoxB which are superior in activity*

*• S. cummioxidans 35Y represents a species of a novel genus*

**Supplementary Information:**

The online version contains supplementary material available at 10.1007/s00253-023-12940-3.

## Introduction

Natural rubber (NR) is present in over 2000 plant species including the rubber tree (*Hevea brasiliensis*). The latter produces a milky liquid (latex) in special tissues, the laticifers, that consists of an aqueous emulsion with poly(*cis*-1,4-isoprene) as a main component (25 to 35% [wt/wt]) besides minor amounts of proteins and other components (Rose and Steinbuchel 2005). NR is an excellent polymer for the synthesis of several goods such as surgical gloves, tyres, toys, and medical devices. During the COVID-19 pandemic, there was a huge increase in the NR waste generated from medical products. Most of the NR waste management methods are based on employing physical or chemical treatments, which have several health and environmental hazards (Arias and van Dijk 2019). NR-degrading microbes (bacteria and fungi) act on the polyisoprene chains and utilize them as a carbon source for their growth. For previous overviews on NR-biodegrading bacteria, see Jendrossek et al. (1997), Rose and Steinbuchel (2005), Warneke et al. (2007), Yikmis and Steinbüchel (2012), Ali Shah et al. (2013), and Chengalroyen and Dabbs (2013). The average time for NR degradation under laboratory conditions by Gram-positive microbes is 6 to 12 weeks; however, the rubber oxygenase of Gram-negative bacterium, *Steroidobacter cummioxidans* strain 35Y (earlier known as *Xanthomonas* sp. strain 35Y (Tsuchii and Takeda 1990; Sharma et al. 2018)), was the first to show ≈60% NR weight loss in only 1 week in in vitro experiments. The complete NR metabolism pathway has been elucidated by a combination of experimental investigations and in silico analysis of the genome of *S. cummioxidans* 35Y (Sharma et al. 2018) as well as in a Gram-positive NR-degrader, namely *Nocardia nova* SH22a (Luo et al. 2014). For bacterial NR degradation, extracellular cleavage of NR into a mixture of small oligoisoprenoids is the first step. In Gram-negative bacteria, this cleavage is carried out by rubber oxygenases, namely RoxA and RoxB, which were originally identified in *S. cummioxidans* 35Y for the first time (Braaz et al. 2004; Jendrossek and Birke 2019). RoxA performs exo-cleavage and RoxB performs endo-cleavage of the polyisoprene molecule. As a result, the products differ with RoxA leading to the formation of (C_15_) oligoisoprenoids as main cleavage product and with RoxB leading to C_20_ or larger oligoisoprenoids (Jendrossek and Birke 2019). The purpose of this review article is twofold: first to review the recent developments that took place with regard to conservation, evolutionary, and functional aspects of the different types of rubber oxygenases and second is to highlight the evolutionary aspects of the bacteria exhibiting rubber degradation potential and harbouring these rubber oxygenases.

## Rubber oxygenases

### RoxA-type rubber oxygenases (RoxAs)

In addition to the RoxA_35Y_, other, at least, partially characterized RoxAs are those of *Haliangium ochraceum* SMP-2 (RoxA_Hoc_), *Corallocoocus coralloides* BO35 (RoxA_Cco_), *Myxococcus fulvus* HW1 (RoxA_Mfu_) (Birke et al. 2013), and *Rhizobacter gummiphilus* NS2 (Kasai et al. 2017) (Birke et al. 2017). RoxAs have two binding motifs (CxxCH) for covalent attachment of the iron-containing cofactor haem thereby classifying them as dihaem* c*-type cytochromes (Jendrossek and Reinhardt 2003). Determination of the RoxA_35Y_ structure revealed a core in RoxA_35Y_ that includes the two haem cofactors and is structurally almost identical to cytochrome* c* peroxidases (Seidel et al. 2013). The C-terminal haem of RoxA_35Y_ is sixfold coordinated (with a Fe^3+^ ion, 4 N-Fe bonds from haem and two axial N-Fe-bonds from conserved main-chain histidine residues) but the N-terminal haem in RoxAs has only one axial ligand (His195) leaving the 6th position open. In RoxA_35Y_ and RoxA_NS21_, a dioxygen molecule is bound to the 6th coordination site of the haem iron (Fe^2+^-O_2_^−^) (Jendrossek and Birke 2019; Seidel et al. 2013). The N-terminal haem and the space above the oxygenated iron builds up the active site that is surrounded by a series of hydrophobic residues (A251, I252, L254, I255, F301, A316, and F317). The dioxygen molecule is stabilized by Phe317 (*S. cummioxidans*) corresponding to Phe302 in *R. gummiphilus* RoxA (Seidel et al. 2013) (Birke et al. 2012). Spectroscopic analysis revealed that the N-terminal active site haem is present in a reduced state (Fe^3+^---O_2_^−^/Fe^2+^--- O_2_ equilibrium) while the C-terminal haem is present in the oxidized Fe^3+^ form (Schmitt et al. 2019, 2010). A dioxygenase cleavage mechanism has been experimentally shown only for RoxA_35Y_ (Braaz et al. 2005) but is likely also true for other RoxAs.

### RoxB-type rubber oxygenases (RoxBs)

The genomes of most Gram-negative NR-degrading bacteria have a second, *roxA*-related gene, coding for a protein sharing 35–40% identity with RoxAs including an N-terminal signal peptide for extracellular proteins and two haem binding motifs (Jendrossek and Birke 2019; Kasai et al. 2017; Sharma et al. 2020). Heterologous expression of these genes of *S. cummioxidans* 35Y *roxB*_*35Y*_) and *R. gummiphilus NS21* (*latA*) (in a *S. cummioxidans* background) and biochemical characterization of the purified proteins revealed that they catalyzed the dioxygen-dependent cleavage of poly(1,4-*cis*-isoprene) and thus represent true rubber oxygenases which have been designated as RoxB_35Y_ and RoxB_NS21_, respectively (Birke et al. 2017; Jendrossek and Birke 2019). The products of RoxBs-catalyzed cleavage (endo-type) of polyisoprene differ from those of RoxAs (exo-type) in that the products are larger subunits (C_20_- or higher oligoisoprenoids, C_25_-, C_30_-, C_35_-, etc.) (Röther et al. 2017). Interestingly, a cooperative effect on the efficiency of cleavage was determined when RoxA and RoxB were present simultaneously in an in vitro polyisoprene latex cleavage assay (Birke et al. 2018). A similar cooperative effect was noticed when RoxA was combined in vitro with Lcp. The produced oligoisoprenoids with different number of isoprene units could be quantitatively isolated via gel permeation chromatography (Röther et al. 2017) or could be produced in enzyme bioreactors (Andler et al. 2020). Polyisoprene-derived oligoisoprenoids with functional groups (aldehyde and keto groups) are interesting compounds and could be useful building blocks in chemosynthetic and biotechnological applications.

### Proteins related to RoxAs and RoxBs (RoxCs)

A comprehensive in silico analysis identified 34 and 53 sequences similar to RoxA and RoxB, respectively (Sharma et al. 2020). A phylogenetic analysis of these Rox homologues revealed three putative clusters, namely ‘RoxBI’, ‘RoxAII’, and ‘RoxAIII and RoxBIII’ (Sharma et al. 2020). The cluster ‘RoxBI’ was formed by RoxB orthologs and the ‘RoxAII’ cluster was formed by RoxA orthologs; however, the third cluster ‘RoxAIII and RoxBIII’ was a mixed cluster formed by both RoxA and RoxB sequences. This third cluster in the phylogenetic tree suggested the emergence of another putative rubber oxygenase (RoxC) in the gamma-proteobacteria. Although RoxCs revealed similarities to either RoxAs or RoxBs, they differed from those in several aspects (for details, see Sharma et al. (2020)). For example, the signature motifs and residues of RoxC are more similar to RoxB as it harboured the CXXCH motif of haem 1, which was different from that of the sequences of the RoxAII cluster (CSXCH). In addition, members of the putative RoxCs missed some of the strictly conserved residues of active rubber oxygenases such as Phe317 or Trp302 (Jendrossek and Birke 2019).

**Fig. 1:**
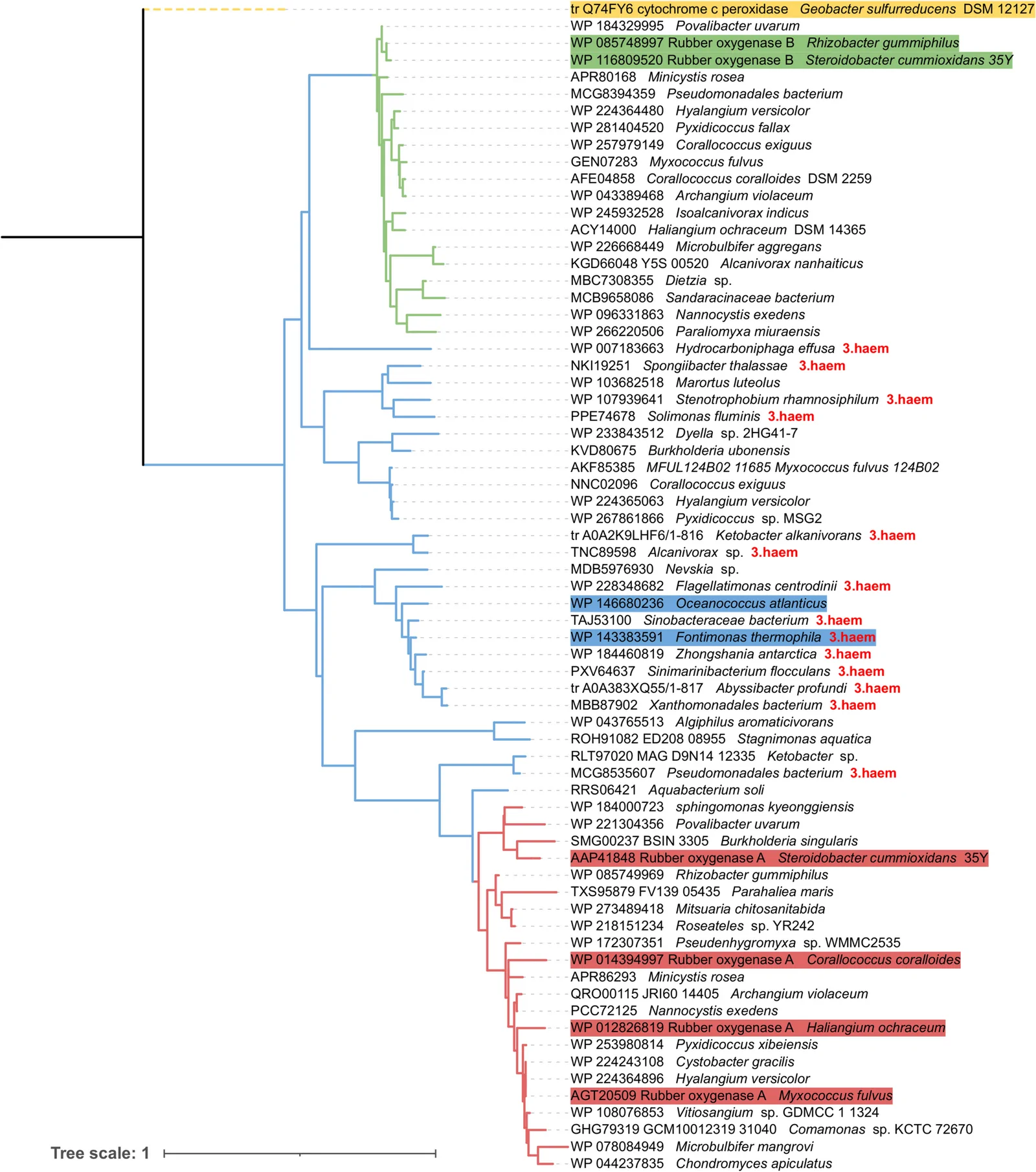
Phylogenetic relationships among orthologs of newly identified rubber oxygenases (RoxA and RoxB). The phylogenetic tree was constructed based on protein sequences of the RoxA and RoxB orthologs by using the neighbor-joining method (Bootstrap 1000). The cytochrome *c* peroxidase protein of *Geobacter sulfurreducens* (DSM 12127) was used as the outgroup (coloured in brown). Solid lines represent the lengths of branches; dotted lines are used to align the tip labels for better visualization. From each of the three groups of the full phylogenetic tree (suppl. Fig. S1), one species of every group was selected as representative. Red, green, and blue solid lines indicate enzymes which were assigned to the RoxA (22 sequences), RoxB (19 sequences), and RoxC (19 sequences) groups, respectively. Enzymes with a red and green background were expressed and biochemically characterized as RoxAs and RoxBs previously; however, enzymes with a blue background were expressed and biochemically characterized but showed no rubber oxygenase activity (unpublished data). Enzymes which contain a third N-terminal haem are indicated by a ‘3.haem’ suffix at the end of their name. Two bacteria of new classes were identified to contain ‘Rox-like’ enzymes. The alpha-proteobacteria *Sphingomonas kyeonggiensis* (indicated by *) and the GC-rich Gram-positive bacterium *Dietzia* sp. (indicated by **) are the first examples of rubber oxygenase orthologs found outside of the beta-, gamma-, and delta-proteobacteria classes

In July 2023, the BLAST search to identify new rubber oxygenases was repeated (see Sharma et al. (2020) for methodology) and the number of identified Rox-related sequences was found to be 458. Briefly, online BLASTP was used with default parameters to search for orthologs of the RoxA (UniProt-ID: Q7X0P3) and RoxB (UniProt-ID: A0A1S6Q8F9) protein sequences of *S. cummioxidans* 35Y against the UniProt (https://www.uniprot.org/, version as of July 2023) and the NCBI database of non-redundant protein sequences (NR) (https://blast.ncbi.nlm.nih.gov/Blast.cgi, version as of July 2023). Orthologs were assigned as sequences with an E-value cutoff of 10^−4^, a threshold of 30% amino acid sequence identity, and 60% coverage of both query and hit. A phylogenetic tree including all 456 Rox orthologues was constructed (Fig. 1 and suppl. Fig. S1) and consisted of three major clusters very similar to that described earlier (Sharma et al. 2020). The first cluster consisted of 117 sequences and was similar to ‘RoxAII’ (RoxAs, red in Fig. 1 and suppl. Fig. S1), the second cluster harboured 98 sequences and was similar to ‘RoxBI’ (RoxBs, green in Fig. 1 and suppl. Fig. S1), and the third largest cluster included 243 sequences and was similar to ‘RoxAIII and RoxBIII’, representing the putative RoxC group (RoxCs, blue in Fig. 1 and suppl. Fig. S1). Interestingly, one of the newly identified RoxA-related proteins was from *Sphingomonas kyeonggiensis*, a species of the alpha-proteobacteria. The identified Rox protein of this species revealed > 70% amino acids identity to RoxA_35Y_ including a putative signal peptide and a MauG motif as well as two haem binding sites and conserved Phe317 and Trp302 residues. Therefore, it is likely that members of the alpha-proteobacteria also have biochemically active RoxAs and will turn out to be able to cleave and to utilize polyisoprene as a carbon source. Similarly, RoxCs are no longer found to be restricted to gamma-proteobacteria. We have identified RoxC orthologues in the members beta-proteobacteria and *Myxococcia*. Another remarkable result was the identification of the first Gram-positive bacterium (*Dietzia* sp.) harbouring a putative *roxB* gene (Fig. 1 and suppl. Fig. S1). This RoxB-like protein, while having two haem binding sites and other RoxB-signatures, has a predicted molecular mass of only ≈50 kDa and lacked a signal peptide sequence. Since the protein sequence started with a serine (instead of methionine) and the DNA sequence of this protein was from a shotgun sequencing project, it is possible that the (true) start of the gene is missing and the resulting ‘complete’ polypeptide will turn out to have the missing RoxB features as well.

Another interesting feature of most RoxC sequences is the presence of a signal peptide cleavage site with a conserved cysteine directly after the predicted cleavage site. This is typical for lipoproteins in which the protein is anchored with a covalently bonded acyl-glycerol to the (outer) membrane. We, therefore, predict that RoxC proteins will turn out to be attached to the (outer) cell membrane. Furthermore, a substantial number of RoxC sequences revealed the presence of an N-terminal domain harbouring a (third) haem binding motif (CxxCH) in addition to the two haem binding motifs of the putative catalytic domain related to RoxAs and RoxBs. (Fig. 1 and suppl. Fig. S1). The different features of the RoxA, RoxB, and RoxC proteins are summarized in Table 1. Unfortunately, no biochemical data for any protein of this third branch was available until recently. The (recent) expression of two selected *roxC* genes (from *Oceanococcus atlanticus* and *Fontimonas thermophilus*) and partial purification of the gene products (RoxC_Oat_, RoxA_Fth_) surprisingly could not demonstrate any rubber-cleaving activity and no polyisoprene latex–dependent oxygen consumption for these proteins (unpublished results). Furthermore, the theoretical isoelectric points (IEPs) of RoxCs generally were strongly acidic (IEP [RoxC_Fth_] 5.1; IEP [RoxC_Oat_] 4.6) while the IEP of RoxA_35Y_ was substantially higher (pH, 7.3). The biological function of RoxCs is therefore currently unknown. The indication for RoxCs as lipoproteins might point to a function in an oxidation of unsaturated fatty acids present in the cell wall of Gram-negative bacteria.

**Table 1:** Properties of rubber oxygenases

Protein/attribute	RoxA_35Y_	RoxB_35Y_	RoxC_Fth_	RoxC_Oat_
Gene length [bp]	2037	2046	2454	2136
Secretion system	Sec	Sec	Sec (lipoprotein)	Sec (lipoprotein)
Signal peptide cleavage site	ALA/L_17_TP	TDA/A_34_TD	LGG/C_21_AG	LVA/C_16_NG
Mw pre-protein [kDa]	74.7	73.8	88.9	77
Mw mature protein [kDa]	71.5	70.3	86.9	75.4
IEP mature (theor.)	7.3	6.3	5.04	4.63
% of arom. AA	11.4	9.9	9	8.9
Total no.: F, Y, W	(24, 30, 20)	(23, 24, 17)	(31, 24, 17)	(26, 20, 16)
Available structures	X-ray (1.80 Å)	Alphafold	Alphafold	Alphafold
*Haem attachment*				
N-terminal	CSACH_195_	CHACH_196_	CFQCH_312_	CFQCH_204_
C-terminal	CASCH_394_	CASCH_395_	CASCH_538_	CASCH_437_
3. haem (N-terminal)	no	no	CRTCH_44_	no
*Axial haem ligands*				
N-terminal	H_195_	H_196_	H_312_	H_204_
C-terminal	H_394_ H_641_	H_395_ H_627_	H_538_ H_776_	H_437_ H_675_
3. haem (N-terminal)	-	-	H _44_ H _98_	-
Fe state ‘as isolated’	Fe^2+^–O_2_/Fe^3+^	Fe^3+^/Fe^3+^	Fe^3+^/Fe^3+^/Fe^3+^	Fe^3+^/Fe^3+^
MauG motif	PYFH_517_NGSVP	PYMH_494_NGSVP	PYDH_673_NGSVP	PYLH_553_ NGSVP
F317 equivalent (according to structural alignment)	F_317_	F_309_	A_474_	A_354_
W302 equivalent (according to structural alignment)	W_302_	V_294_	F_459_	F_339_
Soret max (ox) [nm]	407	404	409	408
Soret max (reduced) [nm]	418	419	418	418
Beta (reduced) [nm]	549, 553	548, 556	551	551
Alpha (reduced) [nm]	521	522, 529	522	522
Bands above 600 nm	No	~ 618	No	No
UV/Vis effect upon addition of CO	Yes	No	No	-
Cleavage product(s)	ODTD	Pattern	No rubber oxygenase activity	No rubber oxygenase activity

*RoxA*_*35Y*_, RoxA from *S. cummioxidans* 35Y; *RoxB*_*35Y*_, RoxB from *S. cummioxidans* 35Y; *RoxC*_*Fth*_, RoxC from *Fontimonas thermophila*; *RoxC*_*Oat*_, RoxC from *Oceanococcus atlanticus*; *ODTD*, 12-oxo-4,8-dimethyltrideca-4,8-diene-1-al; -: feature not present; pattern: mixture of C20, C25, C30,…..and higher oligoisoprenoids

## Evolutionary aspects of NR-degrading enzymes and taxonomic position of strain 35Y

All previously identified RoxA and RoxB homologues belong to the beta-, gamma-, and delta- classes of the phylum *Proteobacteria* (Sharma et al. 2020). Our recent BLAST search indicates the existence of at least one RoxB in alpha-proteobacteria also and RoxC in beta-proteobacteria and *Myxococcia*, in addition to gamma-proteobacteria. Horizontal gene transfer (HGT) is a potential mode of propagation of genes in different taxonomic lineages. Both *roxA* and *roxB* genes showed a number of interclass routes of HGTs among the members of phylum Proteobacteria. The *roxA* and *roxB* genes were found to originate in the delta-proteobacteria and have later propagated to species of the beta- and gamma-proteobacteria (Sharma et al. 2020).

The previous findings suggest that RoxA-, RoxB-, and RoxC-like proteins are (at present) restricted to Gram-negative bacteria (Jendrossek and Birke 2019; Sharma et al. 2020, 2018); however, the recent results show that Gram-positive microbes can also harbour *roxB* genes. The extracellular cleavage of NR in most other Gram-positive bacteria is carried out by the latex clearing protein (Lcp). The so far only example of a Gram-negative microbe with an *lcp* gene (in addition to *rox* genes) is *Solimonas fluminis* HR-BB (Birke and Jendrossek 2019; Sharma et al. 2018). Although a synergistic activity of RoxA (RoxA_35Y_) with respect to RoxB (RoxB_35Y_) and Lcp (from Gram-positive *Streptomyces* sp. K30) was found, however, NR degradation properties of *S. fluminis* HR-BB have not been demonstrated till now. Percentage similarity of Lcp with RoxA and RoxB is 12.1% and 11.6%, respectively, confirming that Rox proteins and Lcp proteins are not related and have evolved independently.

**Fig. 2:**
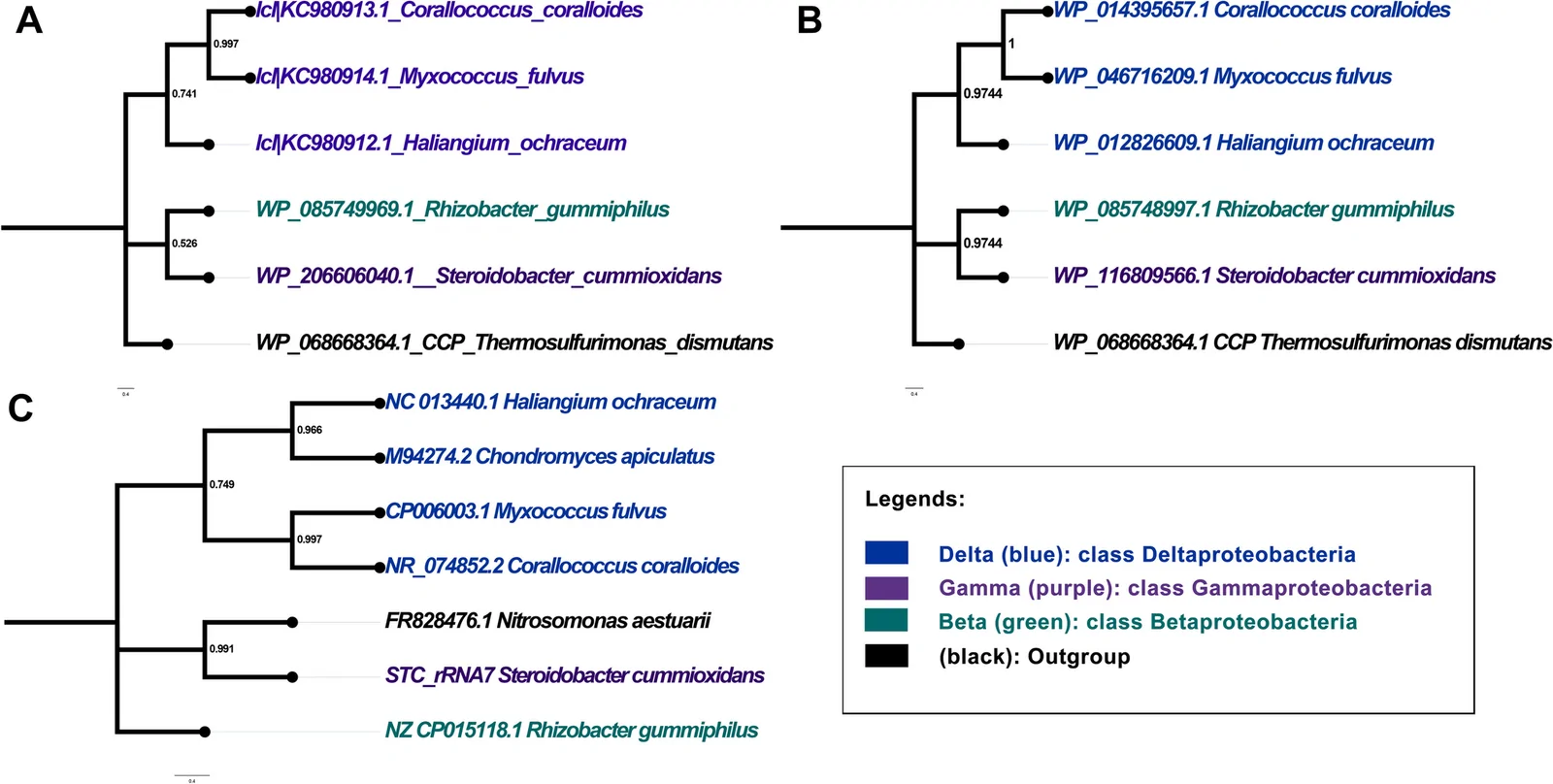
Phylogenetic relationships between *S. cummioxidans* 35Y and the other bacteria, whose RoxA proteins have been found to harbour rubber oxygenase activity, on the basis of **A** RoxA protein, and **B** RoxB protein, and **C** 16S rRNA gene sequences. For insets **A** and **B**, the phylogenetic tree was constructed based on amino acid sequences of the RoxA and RoxB orthologs by using the neighbor-joining method. The cytochrome *c* peroxidase amino acid sequence of *Thermosulfurimonas dismutans* was used as the outgroup. For inset **C**, the phylogenetic relationships between *S. cummioxidans* 35Y was inferred by using the Maximum Likelihood method and Kimura 2-parameter model (Kimura 1980). Initial tree for the heuristic search was obtained automatically by applying Neighbor-Join (Saitou and Nei 1987) and BioNJ algorithms to a matrix of pairwise distances estimated using the Maximum Composite Likelihood (MCL) approach, and then selecting the topology with superior log likelihood value. Bootstrap value (Felsenstein 1985) (resampled 1000 times) is shown in % and values less than 50% are not shown. Evolutionary analyses was performed in MEGA X (Kumar et al. 2018) and the phylogenetic tree was formatted using figtree

A phylogenetic analysis of the microbes harbouring rubber oxygenases revealed that both RoxA_35Y_ and RoxB_35Y_ are remarkably distinct from the other respective orthologues (Sharma et al. 2020). A similar taxonomic analysis using 16S rRNA and *roxA* and *roxB* genes of bacteria whose *roxA* gene products were confirmed to harbour rubber oxygenase activity also reveals a distinct branching of *S. cummioxidans* 35Y from the other microbes (Fig. 2). Besides, the rubber oxygenase degrading activity of *S. cummioxidans* 35Y is known to be superior over other investigated NR-degrading species in its activity leading to a relatively fast NR degradation. This raised significant interest among the researchers to explore the evolutionary and taxonomic aspects of this special microbe. Initial reports on *S. cummioxidans* 35Y termed it as *Xanthomonas* 35Y and considered it as a member of the order *Xanthomonodales* within gamma-proteobacteria (Tsuchii and Takeda 1990). In 2015, Naushad et al. reclassified *Xanthomonodales* and proposed a novel order *Nevskiales* under the class gamma-proteobacteria (Naushad et al. 2015). Subsequently, using a polyphasic taxonomic approach on several features including genomic, physiological, biochemical, and chemotaxonomic traits, three families in the order *Nevskiales*, namely, *Algiphilaceae*, *Sinobactereaceae* (*Solimonodaceae*), and *Salinispharaceae*, were proposed (Naushad et al. 2015; Sharma 2019); however, only the former two families were classified in the NCBI taxonomy database at that time. With the availability of the whole genome sequence of *S. cummioxidans* 35Y in 2018 and the 16S rRNA gene sequences of additional *Steroidobacter* species, the tentative taxonomic positioning of genus *Steroidobacter* was also revised (Sharma 2019) and it became obvious that strain 35Y could not be a species of *Xanthomonas* but represents (at least) a new species of a new genus for which the designation *Steroidobacter cummioxidans* 35Y was chosen (Sharma et al. 2018). An in silico analysis revealed that the genera *Steroidobacter* and *Povalibacter*, which are a part of *Sinobactereaceae*, clustered separately as a sub-clade from the rest of the members of the families *Sinobactereaceae* and *Algiphilaceae*. This led to the proposition of a novel family *Steroidobacteraceae* fam. nov. within the order *Nevskiales* by Sharma (2019) to accommodate three genera, including *Steroidobacter* and *Povalibacter*. The proposal for a novel family *Steroidobacteraceae* fam. nov. was also made by Liu et al. (2019) based on 16S rRNA gene sequence analysis while annotating a novel microbe *Stenotrophobium rhamnosiphilum* (GT1R17T) isolated from a glacier. Recently, genetic-based analysis proposed the new order *Steroidobacterales* which includes the family *Steroidobacteraceae* (Montecillo 2023).

Interestingly, the whole genome sequence–based analysis clearly separated *S. cummioxidans* 35Y from the other species of the genus *Steroidobacter* by its nearly double size of the genome (≈7.9 Mb for 35Y compared to 3.5 Mb for *S. denitrificans*). Although *S. cummioxidans* 35Y exhibits high 16S rRNA gene sequence identity with a few species of genus *Steroidobacter* that would point to be a member of the genus *Steroidobacter*, the analysis of the whole genome sequences ANI (average nucleotide identity) and in silico DDH (DNA-DNA hybridization) could not classify strain 35Y into any of these species (Sharma et al. 2018). This was further supported by POCP (percentage of conserved proteins), ANI, and 271 proteins based core AAI (average amino acid identity) analysis (Sharma 2019). In addition, the morphological and physiological features of *S. cummioxidans* 35Y were found to be different from those of the type species of genus *Steroidobacter* (*S. denitrificans*) (Sharma 2019; Fahrbach et al. 2008). Significant differences of *S. cummioxidans* 35Y to the other species of genus *Steroidobacter* were also identified with respect to the utilization of carbon sources and growth conditions (D. Jendrossek, unpublished data; Sharma 2019)). The chemotaxonomic features of *S. cummioxidans* 35Y were significantly different from the other members of genus *Steroidobacter* (Sharma et al. 2018). These important differences in the physiological, biochemical, and chemotaxonomic and genome-characteristics between *S. cummioxidans* 35Y and the type species of the genus *Steroidobacter* suggest that *S. cummioxidans* 35Y is a species of a novel genus. Based on these differences, *S. cummioxidans* 35Y was suggested to represent the first species of a new genus for which the designation *Aurantibaculum* gen. nov. had been proposed (D. Jendrossek, unpublished data; Sharma 2019). The taxonomic designation of strain 35Y would be *Aurantibaculum cummioxidans* 35Y (golden rod utilizing rubber).

## Microbial consortia–based NR biodegradation

Recently, the use of microbial consortia for NR biodegradation has been attempted by adopting different strategies of using mono-cultures, mixed cultures, natural consortia, synthetic microcosms, or consortia formulated by using enrichment techniques (Veenagayathri and Ahongsangbam 2017; Nawong et al. 2018; Nguyen et al. 2020; Bosco and Mollea 2021; Cheng et al. 2024). It is important to note that a direct comparison of NR biodegradation of individual species and consortia is necessary in the future as the results of the consortia cannot be directly compared with those obtained using pure cultures. In principle it is, however, possible that the use of consortia may be more beneficial than pure cultures in the biodegradation of natural rubber. The use of consortia might also be beneficial to degrade chemically modified (e.g. vulcanized) rubber.

## Summary

The biodegradation of NR in Gram-negative proteobacteria is carried out by two types of rubber oxygenases, namely RoxA and RoxB. These enzymes differ in their catalytic mode of action. However, both these enzymes work in a synergistic manner in the case of *S. cummioxidans* 35Y. A third type of Rox-related proteins (RoxCs) is also present in many Gram-negative microbes, however, at present restricted to species of the beta- and gamma-proteobacteria. The rubber oxygenases of *S. cummioxidans* 35Y appear to be superior thereby leading to a relatively fast NR biodegradation process. The phylogenetic analysis using the whole genome sequence of *S. cummioxidans* 35Y led to the proposal of three families in the order *Nevskiales* including the novel family *Steroidobacteraceae* fam. nov. Within the genus *Steroidobacter*, the physiological properties and the doubled genome size of *S. cummioxidans* 35Y compared with the other species of genus *Steroidobacter* suggest that strain 35Y is a species of a novel genus (*Aurantibaculum* gen. nov.) within the *Steroidobacteraceae*.

## Supplementary Information

Below is the link to the electronic supplementary material.

Supplementary file1 (PDF 4750 KB)

## Data Availability

All data supporting the findings of this study are available within the paper and its Supplementary Information.
