# Supplementary material for: Cleavage of natural rubber by rubber oxygenases in Gram-negative bacteria
Source: Appl Microbiol Biotechnol. 2024 Feb 2;108(1):191. doi: 10.1007/s00253-023-12940-3 (PMC10837239; doi:10.1007/s00253-023-12940-3)
Supplement: Supplementary file 1 — Supplementary file1 (PDF 4750 KB) [file 253_2023_12940_MOESM1_ESM.pdf]

Cleavage of Natural Rubber by Rubber Oxygenases in Gram-negative bacteria.

Tulika Prakash[1#*],Sandhya R. Yadav[1], Marius Bürger[2], Dieter Jendrossek[2]

1: School of Biosciences and Bioengineering, Indian Institute of Technology (IIT), Mandi, HP, India

2: Institute of Microbiology, University Stuttgart, Germany;

#: First Author

***Corresponding author:**

Prof. Tulika Prakash

Professor,

School of Biosciences and Bioengineering,

Indian Institute of Technology (IIT), Mandi, HP, India - 175005

Email: tulika@iitmandi.ac.in

Note: All correspondence concerning this manuscript for bioinformatics and taxonomic aspects may be addressed to T.P (tulika@iitmandi.ac.in) and that for the biochemical aspect on Rox proteins may be addressed to D.J. (dieter.jendrossek@imb.uni-stuttgart.de).

Supplementary Data:

Suppl. Fig. S1: Phylogenetic relationships among orthologs of newly identified rubber oxygenases (RoxA and RoxB). The phylogenetic tree was constructed based on protein sequences of the RoxA and RoxB orthologs by using the neighbor-joining method (Bootstrap 1000). The cytochrome c peroxidase protein of *Geobacter sulfurreducens* (DSM 12127) was used as the outgroup (coloured in brown). Solid lines represent the lengths of branches; dotted lines are used to align the tip labels for better visualization. Red, Green, and Blue solid lines indicate enzymes which were assigned to the RoxA- (117 sequences), RoxB- (98 sequences), and RoxC- (243 sequences) group, respectively. Enzymes with a red and green background were expressed and biochemically characterised as RoxAs and RoxBs previously, however, enzymes with a blue background were expressed, biochemically and showed no rubber oxygenase activity (unpublished data). Enzymes which contain a third Nterminal haem are indicated by a "3.haem" suffix at the end of their name. Two bacteria of new classes were identified to contain "Rox-like" enzymes. The alphaproteobacteria *Sphingomonas kyeonggiensis* (indicated by *) and the GC-rich Grampositive bacteria Dietzia sp. (indicated by **) are the first examples of rubber oxygenase orthologs found outside of the beta-, gamma-, and delta-proteobacteria classes.

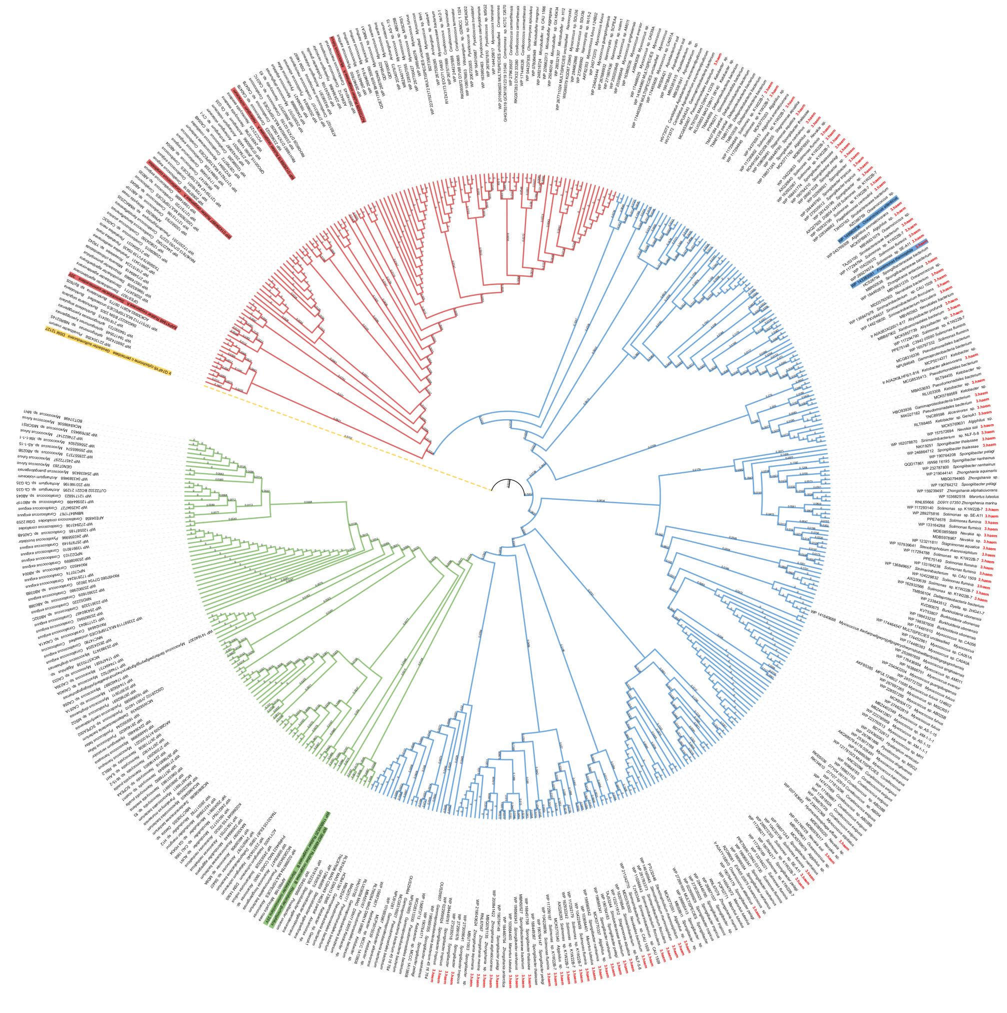

Suppl. Fig. S1
